# Perceptions and misperceptions about lithium: Ar-razi hospital experience

**DOI:** 10.1192/j.eurpsy.2025.1882

**Published:** 2025-08-26

**Authors:** A. Souidi

**Affiliations:** 1 Psychiatry B, Ar-razi hospital, Salé, Morocco

## Abstract

**Introduction:**

Lithium enjoyed its first golden age between 1965 and 1990, but interest in the molecule has not waned, on the contrary, the last 20 years have seen a veritable renaissance in lithium publications. This literature is fuelled by the ongoing exploration of lithium’s unique mood-stabilizing, anti-suicidal and neuroprotective properties, a panoply of properties never before observed in a single molecule. In spite of this, the literature describes little use of lithium, despite ample evidence of its benefits.Our study points the perceptions and misperceptions of lithium and their effects on it use in our hospital.

**Objectives:**

To study and identify the perceptions and misperceptions of doctors at Ar-razi Hospital about lithium, and thus discuss both of efficacy and safety misperceptions through a litterature review. This study will have as a first objective to correct these mispercetions in order to promote a proper care of our patients.

**Methods:**

This is a descriptive study. Data collection was carried out on 50 psychiatrists and trainees. By using an online and handout forms focusing on physicians perceptions and misperceptions of the efficacy and safety of lithium at Ar-razi Hospital in Salé. Jamovi 2.3 was used for data entry and statistical analysis.

**Results:**

our study results were resumed in 2 images. Image 1 is a table that shows the status of our professionnals and also their use of lithum as first or second molecule.

image 2 resumes efficacy perceptions of lithium among our psychiatrists and trainees.

although many other results will be shown by text:

- 58% thinks that Lithium should not be used in women of childbearing age due to teratogenic risk
- 73% have the perception that Lithium should be avoided in elderly patients suffering from a lack of efficacy data and safety concerns.
- 53% thinks that other thymoregulators are safer and should be systematically used in women of childbearing age with bipolar disorder instead of lithium.
- 84% acclaim that lithium treatment is stopped because of it security profile, when 44% of them thinks that hypothyroidism is prevalent as a secondary effect and causes treatment stop.

**Image 1:**

**Figure:**
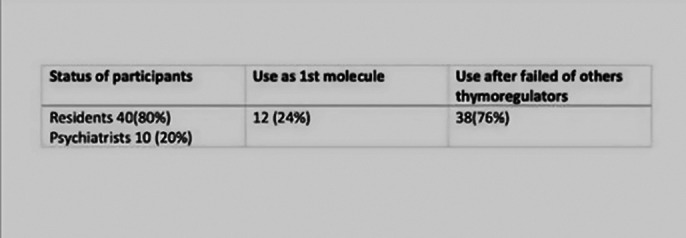

**Image 2:**

**Figure:**
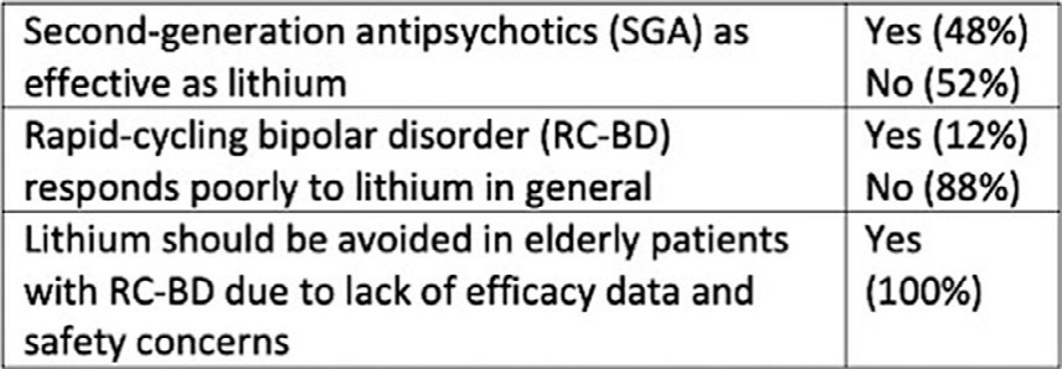

**Conclusions:**

Our study finds that our hospital professionnals have many misperceptions about lithium, especially about it security, which may affect the treatment plan for many patients. This study and it discussion throught the litterature review will change these perceptions, furthermore give the patients a proper care.

Disclosure of new knowledge is essential to refute misconceptions about lithium and enable patients to access its unique therapeutic constellation.

**Disclosure of Interest:**

None Declared

